# Hepatocyte PRMT1 protects from alcohol induced liver injury by modulating oxidative stress responses

**DOI:** 10.1038/s41598-019-45585-2

**Published:** 2019-06-24

**Authors:** Jie Zhao, Abby Adams, Steven A. Weinman, Irina Tikhanovich

**Affiliations:** 1 Department of Internal Medicine, University of Kansas Medical Center, Kansas, United States; 2 Liver Center, University of Kansas Medical Center, Kansas, United States

**Keywords:** Methylation, Alcoholic liver disease, Mechanisms of disease

## Abstract

Protein Arginine methyltransferase 1 (PRMT1) is the main enzyme of cellular arginine methylation. Previously we found that PRMT1 activity in the liver is altered after alcohol exposure resulting in epigenetic changes. To determine the impact of these PRMT1 changes on the liver’s response to alcohol, we induced a hepatocyte specific PRMT1 knockout using AAV mediated Cre delivery in mice fed either alcohol or control Lieber-DeCarli liquid diet. We found that in alcohol fed mice, PRMT1 prevents oxidative stress and promotes hepatocyte survival. PRMT1 knockout in alcohol fed mice resulted in a dramatic increase in hepatocyte death, inflammation and fibrosis. Additionally, we found that alcohol promotes PRMT1 dephosphorylation at S297. Phosphorylation at this site is necessary for PRMT1-dependent protein arginine methylation. PRMT1 S297A, a dephosphorylation mimic of PRMT1 had reduced ability to promote gene expression of pro-inflammatory cytokines, pro-apoptotic genes BIM and TRAIL and expression of a suppressor of hepatocyte proliferation, Hnf4α. On the other hand, several functions of PRMT1 were phosphorylation-independent, including expression of oxidative stress response genes, Sod1, Sod2 and others. *In vitro*, both wild type and S297A PRMT1 protected hepatocytes from oxidative stress induced apoptosis, however S297D phosphorylation mimic PRMT1 promoted cell death. Taken together these data suggest that PRMT1 is an essential factor of liver adaptation to alcohol; alcohol-induced dephosphorylation shifts PRMT1 toward a less pro-inflammatory, more pro-proliferative and pro-survival form.

## Introduction

Alcohol is a leading cause of preventable morbidity and mortality worldwide^1–3^. Chronic alcohol abuse underlies the pathogenesis of alcoholic liver disease (ALD), encompassed by a spectrum of pathologies, ranging from steatosis to more severe forms of liver injury, including alcoholic hepatitis (AH), fibrosis and cirrhosis. AH, an inflammatory condition characterized by infiltration of leukocytes and hepatocellular injury, remains an important contributor to mortality from ALD^3–5^. Alcohol interacts with other causes of liver disease, including hepatitis B and C, and conditions such as diabetes and obesity to increase the risk for developing alcoholic hepatitis, cirrhosis and eventually hepatocellular carcinoma, either synergistically or additively^3,5,6^. The specific mechanisms responsible for ALD development and progression are not fully understood.

Protein arginine methylation is a common posttranslational modification that plays a role in multiple pathways, including cell cycle control, RNA processing, innate immune responses, apoptosis, oxidative stress responses and other processes^7^. PRMT1 is responsible for about 85% of total cellular arginine methylation^8^. PRMT1 catalyses arginine mono- and dimethylation using S-adenosyl methionine (SAM) as a methyl donor. It methylates both histone and non-histone proteins, however many protein targets are not yet defined^9^. Histone methylation includes the H4R3me2a asymmetric methylation mark at histone H4, which promotes gene expression and is a part of the histone code^8,10,11^. As a transcriptional coactivator, PRMT1 is recruited to promoters by many different transcription factors^10–12^. PRMT1 impacts gene transcription and splicing as well as upstream signal transduction^13^.

Under normal conditions, PRMT1 in hepatocytes suppresses proliferation^14^. Here, we found that in alcohol fed mice PRMT1function is altered. PRMT1 does not regulate hepatocyte proliferation and instead its main function is prevention of oxidative stress and promotion of hepatocyte survival. PRMT1 knockout in alcohol fed mice results in a dramatic increase in hepatocyte death, inflammation and fibrosis and increased serum ALT levels suggesting that PRMT1 is protective against alcohol induced liver injury. Our data suggest that alcohol promotes PRMT1 dephosphorylation at S297. Phosphorylation at this site regulates PRMT1 target specificity. We found that this phosphorylation regulates PRMT1 dependent asymmetric di-methyl arginine production, PRMT1-dependent gene expression of cytokines, such as TNFα, FOXO3 target genes BIM and TRAIL, and expression of the Hnf4α gene. On the other hand, several functions of PRMT1 were phosphorylation-independent, including expression of oxidative stress response genes. We found that PRMT1 directly binds to these genes’ promoters and promotes recruitment of p300 acetyltransferase. By this mechanism, PRMT1 protects from oxidative stress induced hepatocytes apoptosis.

## Results

### Hepatocyte-specific PRMT1 knockout mice develop more severe liver injury after alcohol feeding

**Figure 1:**
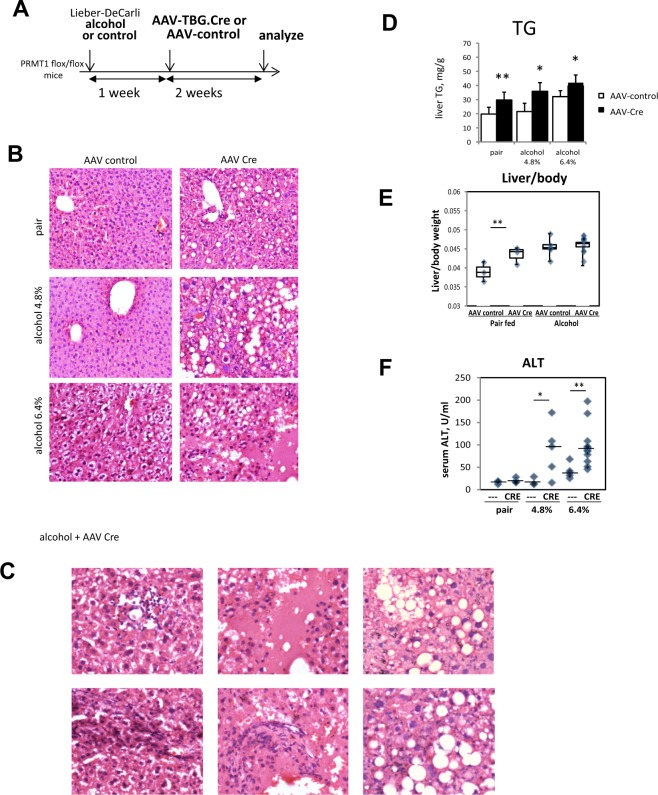
PRMT1 protects liver from alcohol induced liver injury. (**A**) PRMT1 flox/flox mice were for 10 days on liquid diet (control, alcohol 4.8%, or alcohol 6.4%) and received AAV-TBG.control or AAV-TBG.CRE virus (1 × 10^11^ gc/mouse). Mice were left on corresponding liquid diet for 2 more weeks. N = 4–8 mice per group **(B)** H&E staining of liver sections from 6 groups. **(C)** Examples of H&E staining of liver sections from mice fed alcohol diet and received AAV-Cre showing inflammation, hepatocyte death and fibrosis. (**D)** Liver triglyceride levels (TG) in livers of mice. Data are presented as mean ± SD, N = 4–8 mice per group, *p < 0.05 control vs Cre. (**E)** Liver/body weight ratio and (**F**) serum ALT levels in these mice. N = 4–8 mice per group, *p < 0.05, **p < 0.01.

To test the role of PRMT1 in alcohol induced liver injury, we used a hepatocyte specific PRMT1knockout mouse model described before^14^. PRMT1 floxed mice were put on alcohol (6.4% or 4.8%) or control Lieber-DeCarli liquid diet for one week and then injected with AAV-TBG.CRE or AAV-TBG.control vectors (Fig. 1A). Two weeks after AAV injections livers of the mice were analysed. Figure 1B shows representative images of H&E staining. PRMT1 knockout mice on control liquid diet (pair-fed) showed a mild steatosis phenotype. PRMT1 knockout mice on alcohol showed an increase in fat accumulation in the liver as well as an increase in hepatocyte death, liver inflammation and fibrosis, which was more dramatic in the 6.4% alcohol group (Fig. 1C). We confirmed that PRMT1 knockout resulted in an increase in triglyceride content in the livers of both pair-fed and alcohol-fed mice (Fig. 1D).

We found that AAV-Cre administration resulted in an increase of liver/body weight ratio in the pair-fed group. This is consistent with previously published data on the role of PRMT1 in hepatocyte proliferation^14^. However, in the alcohol fed group AAV-Cre did not result in a liver/body ratio change despite an increase in TG content (Fig. 1E).

**Figure 2:**
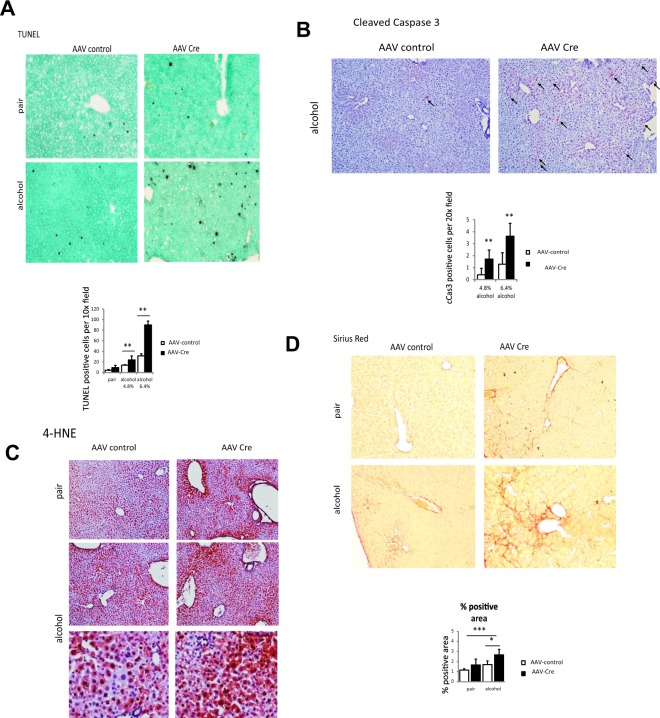
PRMT1 protects liver from oxidative stress and hepatocyte death. (**A**) Representative images of TUNEL staining. Diagram below shows number of positive cells per low magnification field. Data are presented as mean ± SD, N = 3–4 mice per group, **p < 0.01; **(B)** Representative images of immunohistochemistry staining using anti-cleaved Caspase 3 antibodies. Diagram below shows average number of positive cells per field. Data are presented as mean ± SD, N = 3–4 mice per group, **p < 0.01. (**C**) Representative images of 4-HNE staining in livers of the mice as in Fig. 1A. (**D)** Representative images of Sirius Red staining. Diagram below shows average %positive area. Data are presented as mean ± SD, N = 3–4 mice per group, *p < 0.05, ***p < 0.001.

We analysed serum ALT levels in these animals to assess the liver injury. We found that wild type animals did not get an ALT elevation with 4.8% alcohol, while ALT was elevated 3-fold over baseline with 6.4% alcohol. However, PRMT1 knockout mice had an ALT increase of 10-fold in both alcohol groups but not in pair fed animals (Fig. 1F). We confirmed an increase in hepatocyte death using TUNEL staining of PRMT1 wild type and knockout mouse livers. We found higher number of TUNEL positive cells in PRMT1 knockout mice fed alcohol (Fig. 2A). We found that PRMT1 knockout mice have an increase in the number of cleaved caspase 3 positive cells (Fig. 2B), suggesting that PRMT1 knockout in hepatocytes results in an increase in hepatocyte apoptosis. Interestingly, we found that that PRMT1 knockout mice have higher oxidative stress in the liver. We used 4-HNE as a marker of oxidative stress and found that alcohol feeding resulted in a mild increase of 4-HNE in wild type mice. In contrast, knockout mice show a dramatic increase in 4-HNE staining both in pair fed and in alcohol fed groups (Fig. 2C).

Additionally, we found that knockout mice had higher levels of Sirius Red staining (Fig. 2D). Taken together these data suggest that hepatocyte PRMT1 is a protection factor against alcohol induced liver injury in mice.

### Hepatocyte-specific PRMT1 knockout mice have higher oxidative stress levels in the liver

**Figure 3:**
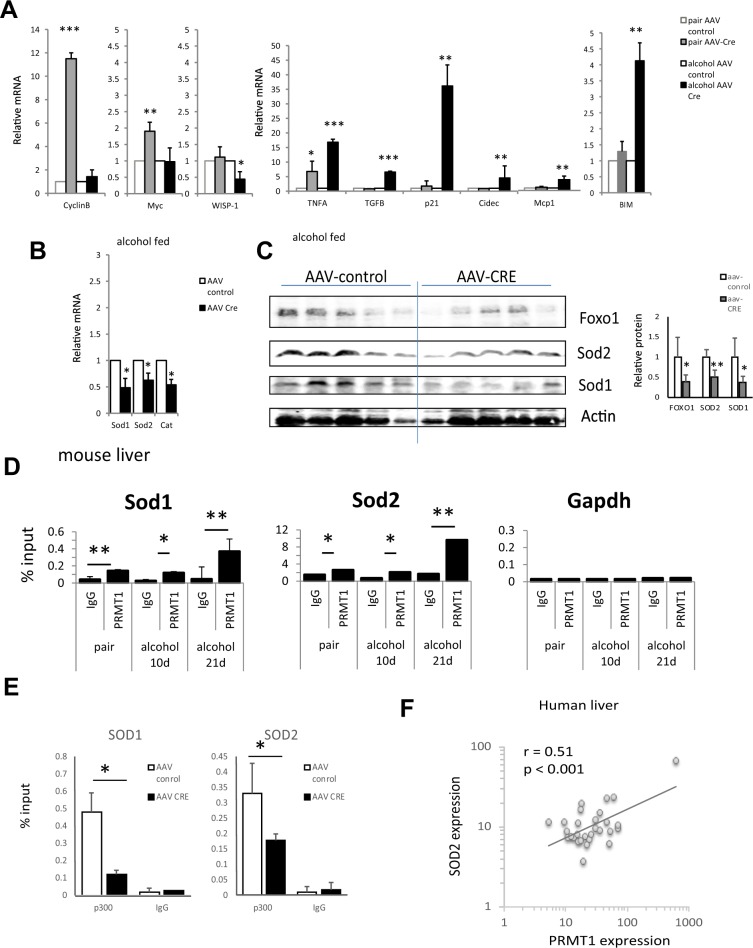
Alcohol alters PRMT1 function in the liver. (**A)** Relative liver mRNA levels in mice fed alcohol or control liquid diet normalized to AAV-control mRNA as in Fig. 1; Data are presented as mean ± SD, N = 4–8 mice per group, *p < 0.05, **p < 0.01, ***p < 0.001 Cre vs control; (**B)** Relative liver mRNA levels in mice fed alcohol (6.4%) normalized to AAV-control mRNA; Data are presented as mean ± SD, N = 6–8 mice per group, *p < 0.05 Cre vs control; (**C)** Western blot analysis of protein levels in these mice. Actin B is used as a loading control. N = 5 mice per group. Densitometry analysis is presented on the right. Data are presented as mean ± SD, *p < 0.05, **p < 0.01 Cre vs control. (**D)** Chromatin immunoprecipitation using anti-PRMT1 or IgG as a negative control from livers of the mice fed control (pair) or alcohol liquid diet for 10 days or 21 days. Data are presented as mean percent of input ± SD. N = 3. *p < 0.05, **p < 0.01. (**E)** Chromatin immunoprecipitation using anti-p300 or IgG as a negative control from livers of the mice fed alcohol liquid diet and received AAV-control or AAV-Cre vectors as in Fig. 1. Data are presented as mean percent of input ± SD. N = 3. (**F**) Correlation between PRMT1 expression and SOD2 expression in human livers from N = 41 liver donors.

To evaluate the pathways involved in PRMT1-dependent regulation of alcohol sensitivity we analysed whole liver mRNA of wild type and PRMT1 knockout mice fed alcohol or control liquid diet (Fig. 3A). We found that PRMT1 knockout in pair fed animals resulted in an increase of proliferation-associated genes CyclinB1 and c-Myc, suggesting that PRMT1 suppresses proliferation under normal conditions. These data are consistent with our previous observation in chow fed mice^14^. In contrast, in alcohol fed mice, PRMT1 knockout did not result in an increase of proliferation-associated genes. Instead knockout mice had higher expression of *Tnfa*, *Tgfb*, *Cidec*, *Mcp1, Cdkn1a* (p21), and *Bcl2l1* (BIM) genes associated with inflammation, fibrosis, lipid accumulation, cell cycle arrest and apoptosis.

To find specific pathways regulated by PRMT1 in alcohol fed mice we analysed liver mRNA of wild type and knockout mice fed alcohol using a PCR-array. We found that one of the top downregulated pathways in knockout mice is the oxidative stress response pathway (Table 1). Specifically, knockout mice have reduced mRNA expression of *Sod1*, *Sod2* and *Cat* (Catalase) (Fig. 3B). We confirmed that the gene expression changes result in changes in protein abundance. We found that knockout mice have reduced protein levels of SOD1, SOD2 and FOXO1 (Fig. 3C) consistent with mRNA results.

**Table 1:** GO term enrichment in top upregulated and down regulated genes in PRMT1 knockout mice fed alcohol.

GO biological process complete	P-value	GO biological process complete	P-value
Downregulated genes		Upregulated genes	
response to drug (GO:0042493)	1.50E-17	regulation of cell cycle	7.87E-16
negative regulation of apoptotic process (GO:0043066)	7.55E-13	positive regulation of protein modification process	3.07E-14
negative regulation of programmed cell death (GO:0043069)	9.78E-13	regulation of cell cycle process	9.95E-14
response to oxygen-containing compound (GO:1901700)	1.99E-12	regulation of protein modification process	2.43E-13
negative regulation of cell death (GO:0060548)	4.43E-12	regulation of protein phosphorylation	2.49E-13
regulation of reactive oxygen species metabolic process (GO:2000377)	8.15E-12	regulation of mitotic cell cycle	7.36E-13
response to chemical (GO:0042221)	9.17E-12	regulation of phosphorylation	7.59E-13

Next, we aimed to determine how PRMT1 regulates SOD1 and SOD2 expression. We found that PRMT1 binds *Sod1* and *Sod2* promoters both in pair fed and in alcohol fed mouse livers (Fig. 3D). We found that PRMT1 is necessary for p300 recruitment to *Sod1* and *Sod2* promoters (Fig. 3E). Additionally, we found that PRMT1 dependent regulation of SOD2 gene expression is relevant in humans. We found a significant correlation between PRMT1 expression and SOD2 expression in normal human liver specimens (Fig. 3F).

### Alcohol promotes PRMT1 dephosphorylation at S297 which results in reduced ability to induce protein methylation

Data presented in Fig. 3 suggest that PRMT1 activity is altered by alcohol. This is consistent with the previous observation *in vitro* and *in vivo* that PRMT1-dependent protein arginine methylation levels were reduced in alcohol fed mice, suggesting that alcohol inhibits PRMT1 activity without affecting its protein levels^14,15^.

**Figure 4:**
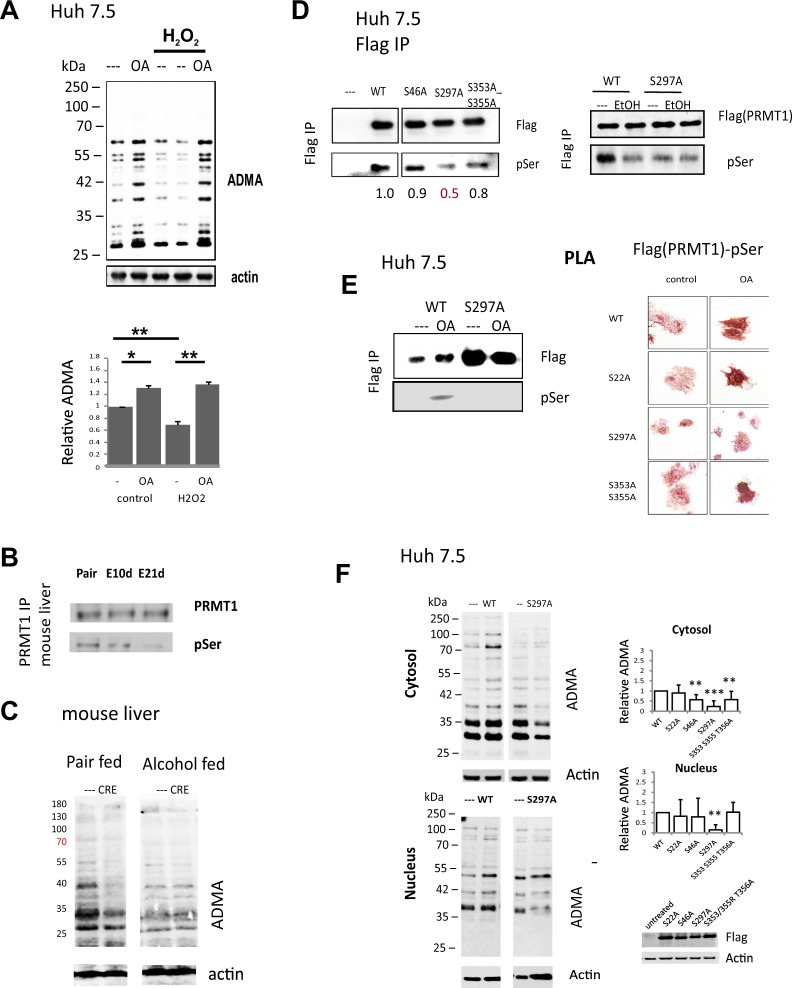
Alcohol promotes PRMT1 dephosphorylation. **(A)** Representative image of western blot analysis of ADMA levels in Huh 7.5 cells treated with 100 µM hydrogen peroxide for 24 hours and PP2A inhibitor 2 nM Okadaic acid (OA) for 8 hours where indicated. Bottom. Densitometry analysis of N = 3 independent experiments. **p < 0.01. (**B)** PRMT1 was immunoprecipitated from mouse liver extracts of mice fed either control liquid diet (pair) or alcohol liquid diet for 10 days or 3 weeks (E10d or E21d). Representative western blot analysis of immunoprecipitated PRMT1 protein using anti-PRMT1 and anti-phospho-serine antibodies. (**C)** Representative image of western blot analysis of ADMA levels in liver sections of mice as in Fig. 1. (**D)** Western blot analysis of PRMT1 wild type or mutants purified from Huh 7.5 cell treated with 50 mM ethanol where indicated using anti-Flag and anti-phospho-serine antibodies. (**E)** Left. Western blot analysis of PRMT1 wild type or mutants purified from Huh 7.5 cells untreated or treated with Okadaic acid (OA) where indicated using anti-Flag and anti-phospho-serine antibodies. Right. Representative images of proximity ligation assay in Huh7.5 cells transfected with Flag-PRMT1 wild type of the mutants and treated with Okadaic acid (OA) where indicated using anti-Flag and anti-phospho-serine (MAPK CDK substrate specific) antibodies. (**F)** Representative image of western blot analysis of ADMA levels in nuclear and cytosolic fractions of Huh 7.5 cells untransfected or transfected with PRMT1 wild type or mutants. Right. Densitometry analysis of N = 3–5 independent experiments. **p < 0.01, ***p < 0.001. ADMA was normalized to the level of ADMA in PRMT1 wildtype transfected cells, 0 – level in untransfected cells.

We aimed to find the mechanism of this alcohol induced PRMT1 activity change. PRMT1 activity is controlled by its phosphorylation and PP2A-mediated dephosphorylation^16^. PRMT1 can be dephosphorylated by PP2A as a result of HCV infection or alcohol treatment^15^. Dephosphorylated PRMT1 is less enzymatically active. We confirmed this mechanism by treating Huh 7.5 cells with okadaic acid (OA), a PP2A inhibitor. OA acid treatment resulted in an increase of PRMT1 phosphorylation and as a result, an increase of PRMT1 activity and cellular protein methylation levels (Fig. 4A). Interestingly hydrogen peroxide treatment, known to activate PP2A, resulted in PRMT1 inhibition, i.e. reduced cellular protein methylation (Fig. 4A). OA treatment restored protein-ADMA levels to the level in untreated cells (Fig. 4A).

Consistent with previous observations we found that alcohol feeding results in reduced PRMT1 phosphorylation (Fig. 4B), which corresponds to reduced protein methylation levels in livers of these mice^14^. We immunoprecipitated PRMT1 from livers of wild type mice fed alcohol or control liquid diet for 10 days or 21 days. We found that alcohol specifically reduced PRMT1 serine phosphorylation but not threonine phosphorylation (not detected) (Fig. 4B). Consistent with the idea that PRMT1 has low activity after alcohol, PRMT1 knockout resulted in reduced liver protein methylation in pair fed but not in alcohol fed mice (Fig. 4C). Taken together these data suggest that alcohol dephosphorylates PRMT1, which makes it enzymatically inactive.

We created PRMT1 S->A mutants for all known PRMT1 phosphorylation sites^17^ to identify functionally relevant site(s) of phosphorylation. We screened these mutants for presence of pSer, ability to promote protein methylation in cells, and change of pSer levels in the presence of okadaic acid. We found that an S297A mutant was less phosphorylated than wild type protein (Fig. 4D). Unlike wild type protein it was not dephosphorylated by alcohol treatment (Fig. 4D). We purified wild type and mutant protein from Huh 7.5 cells untreated and treated with okadaic acid. In contrast to the wild type protein, the S297A mutant did not increase its phosphorylation levels in the presence of okadaic acid (Fig. 4E). Similarly, we assessed PRMT1 wild type and mutant phosphorylation by PLA assay. PLA signals (red-brown dots) were present in cells analysed using a combination of anti-Flag and anti-pSer antibodies. Following treatment with phosphatase inhibitor, signal intensity was increased for wild type, S22A and S353AS355A mutants, but not the S297A mutant (Fig. 4E). Finally, overexpression of the S297A mutant failed to increase ADMA-modified proteins (Fig. 4F). Taken together these data suggest that S297 phosphorylation is a PP2A-dependent phosphorylation site and the phosphorylation at this site regulates PRMT1 protein methylation activity.

**Figure 5:**
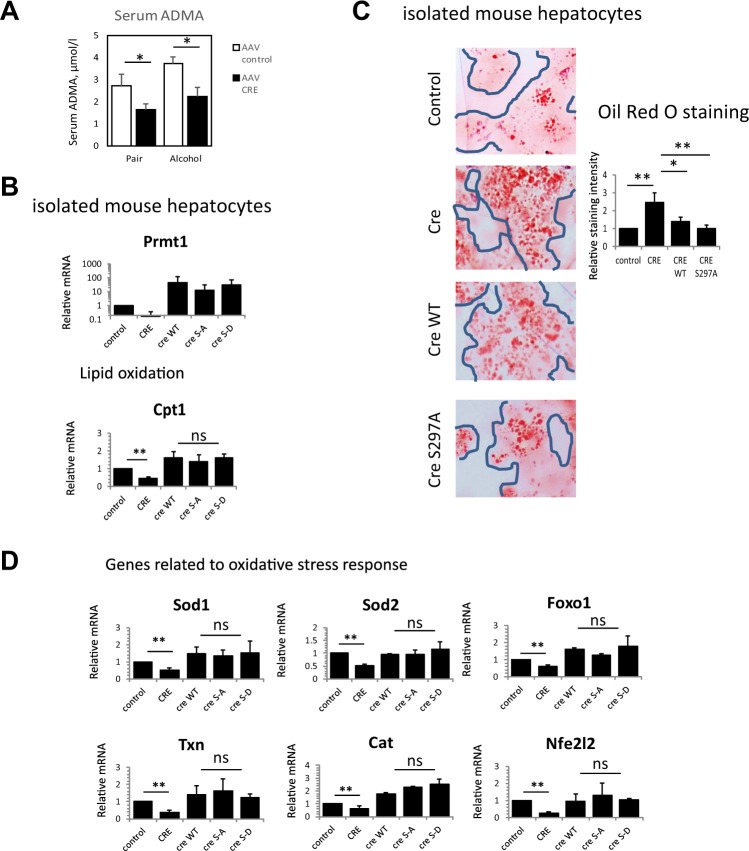
Phosphorylation independent functions of PRMT1. **(A)** Serum ADMA levels in wild type and hepatocyte specific knockout mice as in Fig. 1. Data are presented as mean ± SD, N = 4–8 mice per group, *p < 0.05 control vs Cre. (**B–D)** Hepatocytes were isolated from PRMT1 floxed mice and treated with Ad-Cre to induce PRMT1 knockout or Ad-control vectors. PRMT1 wild type or S297A and S297D mutants were expressed in PRMT1 knockout hepatocytes to assess the function of S297 phosphorylation. (**B)** Relative mRNA expression in these hepatocytes from N = 4–8 independent experiments, data are presented as mean ± SD, **p < 0.01, ns-not significant (**C)** Hepatocytes as in A. were stained with Oil Red O. Right. Relative staining intensity from N = 3 independent experiments, *p < 0.05, **p < 0.01. (**D)** Relative mRNA expression of oxidative stress response genes in isolated hepatocytes as in A. Data are presented as mean ± SD, N = 4–8 independent experiments, **p < 0.01, ns-not significant.

In addition to methylation of protein arginine, PRMT1 also generates free asymmetric dimethyl arginine which can function as an inhibitor of NOS enzymes. We measured serum ADMA levels in wild type and hepatocyte specific knockout mice and found that ADMA levels are equally decreased in the knockouts in both pair fed and alcohol fed groups (Fig. 5A). Thus, in contrast to protein arginine methylation, generation of free ADMA was not affected by alcohol feeding. That suggests that ADMA generation is not controlled by S297 phosphorylation and is thus phosphorylation independent.

### PRMT1 regulates lipid oxidation and oxidative stress response genes independently of S297 phosphorylation

To assess how PRMT1 phosphorylation can affect its function we focused on PRMT1 target genes relevant to the phenotype of PRMT1 knockout mice. To examine how these genes are regulated by PRMT1 we isolated mouse hepatocytes from PRMT1 floxed mice and treated them with Ad-Cre or control vector; additionally, we re-expressed PRMT1 wild type, S297A or S297D in knockout hepatocytes to assess the role of phosphorylation (Fig. 5B).

As mentioned above PRMT1 knockout results in lipid accumulation in the liver (Fig. 2D). This change was not dependent on presence of alcohol, suggesting that it is not dependent on PRMT1 phosphorylation status. Based on PCR array data, lipid accumulation was associated with reduced expression of Cpt1.

We analysed Cpt1 gene expression in wild-type primary hepatocytes, PRMT1 knockout hepatocytes and knockout hepatocytes transfected with either wild type PRMT1or its phosphorylation mutants. PRMT1 knockout resulted in about a 50% reduction of Cpt1 levels. PRMT1 wild type or mutant proteins were equally able to restore Cpt1 levels (Fig. 5B). We assessed lipid accumulation in these hepatocytes using Oil Red O staining after 48 hours of PRMT1 protein expression. We found that PRMT1 knockout results in lipid accumulation and both wild type and S297A PRMT1 restore lipid levels back to baseline (Fig. 5C).

Similarly, PRMT1 regulates oxidative stress response genes such as Sod1 and Sod2. Consistent with an increase in oxidative stress both in pair fed and in alcohol fed mice (Fig. 2D), this function of PRMT1 is phosphorylation independent.

PRMT1 knockout resulted in 40–60% reduction of the oxidative stress response genes *Sod1, Sod2, Txn1* (Thioredoxin 1), *Foxo1, Cat, and Nfe2l2* (NRF2). Regulation of these genes’ expression was not dependent on S297 phosphorylation (Fig. 5D).

### Phosphorylation dependent functions of PRMT1

**Figure 6:**
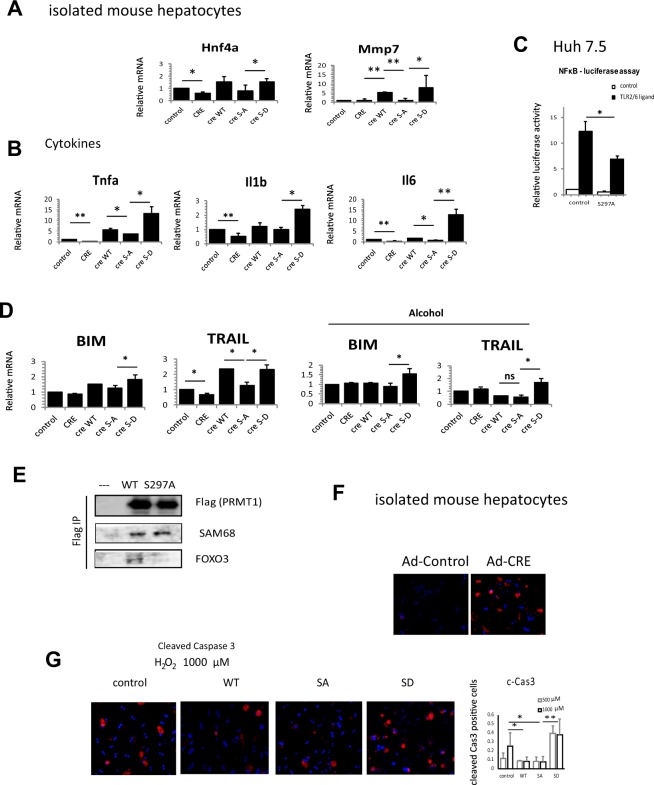
PRMT1 dephosphorylation is required for protection from oxidative stress. **(A)** Hepatocytes were isolated from PRMT1 floxed mice and treated with Ad-Cre to induce PRMT1 knockout or Ad-control vectors. PRMT1 wild type or S297A and S297D mutants were expressed in PRMT1 knockout hepatocytes to assess the function of S297 phosphorylation. (**A**,**B**) Relative mRNA expression in these hepatocytes from N = 4–8 independent experiments, data are presented as mean ± SD, **p < 0.01. (**C)** Relative luciferase activity in Huh 7.5 control or expressing S297A PRMT1 in the presence or absence of FSL-1 from N = 3 independent experiments, data are presented as mean ± SD, *p < 0.05. (**D)** Relative mRNA expression of apoptosis related genes as in A. from N = 4–8 independent experiments, data are presented as mean ± SD, *p < 0.05. (**E)** Wild type or S297A PRMT1 was immunoprecipitated using anti-Flag affinity resin. Huh 7.5 not expressing PRMT1 was used as a negative control. Co-immunoprecipitated proteins were analysed by western blot analysis using anti-Flag (PRMT1), anti-FOXO3 or anti-SAM68 antibodies. (**F)** Hepatocytes were isolated from PRMT1 floxed mice and treated with Ad-Cre to induce PRMT1 knockout or Ad-control vectors. Cells were treated with H_2_O_2_ for 24 h and stained for cleaved-Caspase 3 to detect apoptotic cells. (**G)** Hepatocytes expressing PRMT1 wild type, S297A, S297D or control vector were treated with 0.5 or 1 mM H_2_O_2_ for 24 h and stained for cleaved-Caspase 3 to detect apoptotic cells. Right. Average number of cCas3 positive cells (of total number of cells) in N = 3 independent experiments, *p < 0.05.

We also identified a number of PRMT1 dependent pathways that depend on S297 phosphorylation of PRMT1. S297A, a mutant designed to simulate the dephosphorylated situation after alcohol, was less efficient than either WT or S297D in inducing expression of HNF4α and MMP7 (Fig. 6A).

Phosphorylation is important for PRMT1 dependent cytokine gene expression. We found that the phosphomimetic S297D form showed 2-fold (IL1β), 4-fold (TNFα) and 10-fold (IL6) greater ability to induce cytokine gene expression than the S297A mutant (Fig. 6B). Consistent with that we found that S297A PRMT1 expression in Huh 7.5 cell resulted in reduced NF-κB reporter luciferase activity in the presence of the TLR2/6 ligand FSL-1 (Fig. 6C).

Finally, S297A has reduced ability to induce expression of the FOXO3 target genes BIM and TRAIL (Fig. 6D). In the presence of alcohol, which results in dephosphorylation of WT PRMT1 we found that both WT and S297A failed to induce BIM and TRAIL. In contrast the S297D phosphomimic PRMT1 promoted expression of both genes. Consistent with above results we found that PRMT1 KO in untreated hepatocytes (Fig. 6D, compare control and CRE) resulted in reduced TRAIL expression. However, in alcohol treated hepatocytes PRMT1 KO did not alter TRAIL expression (Fig. 6D). Interestingly we found that the above difference in PRMT1 target specificity corresponded to changes in PRMT1-FOXO3 binding. We found that S297A PRMT1 has reduced ability to bind FOXO3 compared to wild type protein (Fig. 6E). In contrast, binding to another PRMT1 target, SAM68, was not reduced.

Next, we tested the relevance of these findings for protection from oxidative stress induced apoptosis. We isolated mouse primary hepatocytes from PRMT1 floxed animals and treated them *in vitro* with Ad-Cre vector or Ad-control vector. Resulting wild type and knockout hepatocytes were treated with 500 µM H_2_O_2_ and stained with anti-cleaved-caspase 3 antibodies (Fig. 6F). We found that PRMT1 knockout hepatocytes are more sensitive to oxidative stress induced caspase 3 activation (Fig. 6F).

Next, we overexpressed PRMT1 wild type and S297A or S297D mutants and treated hepatocytes with 500 or 1000 µM H_2_O_2._ We found that S297A was as efficient as the wild type protein in protecting hepatocytes from oxidative stress induced caspase-3 activation (Fig. 6G) consistent with protective role of dephosphorylated PRMT1 (S297A cannot be phosphorylated and WT is dephosphorylated in response to oxidative stress). In contrast S297D PRMT1 expression resulted in an increase in oxidative stress induced caspase-3 activation, suggesting that PRMT1 dephosphorylation is an important mechanism of protection from oxidative stress.

## Discussion

PRMT1 regulates multiple aspects of liver biology including cell proliferation^14^, fatty acid metabolism^18^, glucose metabolism^19^, innate immune response^20^, oxidative stress response and apoptosis^15^. It has multiple protein targets including transcription factors (FOXOs, c-Myc, NRF2, HNF4α, p53, β-Catenin, NF-κB, Gli), signalling molecules (ASK, TRAF6, EGFR) and histones (H4, H3)^8,10–12,20–31^. These targets often have opposing roles in the pathways discussed above. Thus, we hypothesized that a change of PRMT1’s function could be a simple mechanism to coordinate changes, both positive and negative, in multiple pathways necessary for response to environmental challenges such as alcohol exposure.

The striking finding of this study is that PRMT1 plays an important role as an alcohol protection factor. While knocking out PRMT1 in hepatocytes has little effect under control conditions, the knock out mice were greatly sensitized to alcohol with increased oxidative stress, elevated serum ALT, increase hepatocyte death and increased lipid accumulation. Interestingly, PRMT1 appeared to have different functions depending on whether or not the mice were on alcohol containing diets. We found that alcohol promoted de-phosphorylation of PRMT1, and this resulted in a change in PRMT1’s main function in the liver from a suppressor of hepatocyte proliferation to a promoter of survival in response to oxidative stress. These different functions of PRMT1 after alcohol exposure depended on its dephosphorylation to different degrees. While cytokine production, BIM, TRAIL and MMP7 expression were dependent on phosphorylation status, control of the oxidative stress response was phosphorylation-independent and, based on our previous work^31^, likely mediated by direct promoter binding and p300 recruitment to Sod1 and Sod2 promoters.

Very little is known about factors that regulate PRMT1 activity and substrate specificity. There is evidence that PRMT1 can be regulated through alternative splicing that changes the protein’s nuclear/cytosolic localization and substrate binding^32,33^. PRMT1 phosphorylation at Y299 is common in tumours and was shown to alter PRMT1 target specificity^34^. PRMT1 activity was shown to be regulated though dephosphorylation by serine/threonine phosphatase PP2A, but the sites of phosphorylation have not been described^16^. Here we found that S297 is a PP2A-dependent phosphorylation site which controls PRMT1’s ability to generate protein arginine methylation. S297 is located within the THW domain of PRMT1, one of the protein substrate binding sites. The THW domain is present in all the PRMTs and has previously been reported to control the ability to make dimethyl arginine (DMA) in PRMT7 and PRMT6^35,36^. Other reports indicated that mutations of human PRMT9 in the THW loop, shifted its product specificity from DMA toward MMA (monomethyl arginine)^37^.

We found that several of PRMT1’s functions are controlled by its phosphorylation at S297. First, the dephosphorylated mimic, S297A PRMT1, was unable or less able to induce cytokine production (*Tnfa, Il1b, Il6*). These data agree with reports that have identified PRMT1 arginine methyltransferase activity as a factor that is required for optimal NF-κB activation^10^. In addition, phosphorylation was required for HNF4α expression regulation. In this case, the regulation is mediated via promoter histone arginine methylation and possibly by arginine methylation of HNF4α itself^12,14^. Interestingly we found that S297 phosphorylation was required for PRMT1-dependent expression of *Mmp7*, a beta-Catenin target gene. MMP-7 is an important matrix metalloproteinase involved in liver fibrosis and cancer metastasis^38^. This function of PRMT1 might be important in further progression of liver disease induced by alcohol.

Finally, PRMT1 phosphorylation was necessary for FOXO3 binding and induction of FOXO3 target genes BIM and TRAIL. Previously we reported that in the presence of alcohol, FOXO3 specifically promotes expression of pro-apoptotic genes, including BIM and TRAIL^39^. Here we found that PRMT1 phosphorylation is a finetuning mechanism of regulation of FOXO3-dependent transcription. PRMT1 phosphorylation is necessary for PRMT1-FOXO3 binding, which is required for FOXO3 stability and activity^15^. Dephosphorylated PRMT1, as a result, is unable to promote BIM and TRAIL expression.

The loss of PRMT1 under alcohol conditions when it is dephosphorylated does not change protein methylation levels, yet it still causes changes in antioxidant and cell death responses. This suggests that PRMT1 has functions independent of its enzymatic activity and may be mediated by direct target promoter binding which we observed for *Sod1* and *Sod2* genes. This is similar to other epigenetic regulators that have both enzymatic activity dependent and independent functions. For example, JMJD3, a well-known histone lysine demethylase has demethylase independent functions as well^40,41^, and the lysyl-oxidases LOXL1 and LOXL2 have enzymatic activity-independent functions in EMT^42,43^.

Another function of PRMT1 we found to be phosphorylation independent is ADMA generation. PRMT1 is a major source of the NOS inhibitor ADMA. We found that hepatocyte PRMT1 makes about one third of serum ADMA, and alcohol does not affect this function. That suggests that ADMA generation is not controlled by S297 phosphorylation, i.e. phosphorylation independent. These data suggest that reduced ADMA in knockout mice fed alcohol can lead to an increase of NO levels in hepatocytes and together with increased superoxide generation (due to low Sod1 and Sod2 levels), this leads to peroxynitrite formation that exacerbates the hepatocyte death and liver injury^44^. The relative contribution of this mechanism to the phenotype of PRMT1 knockout mice fed alcohol is a subject for future studies.

Our data suggest that PRMT1-dependent oxidative stress response gene regulation is relevant in humans as well. We found that the PRMT1 level varies among healthy individuals and correlates with SOD2 expression in the liver. These data suggest that individuals with low PRMT1 and as a result low SOD2 levels can be more susceptible to alcohol induced liver injury. Further studies are necessary to confirm this hypothesis.

**Figure 7:**
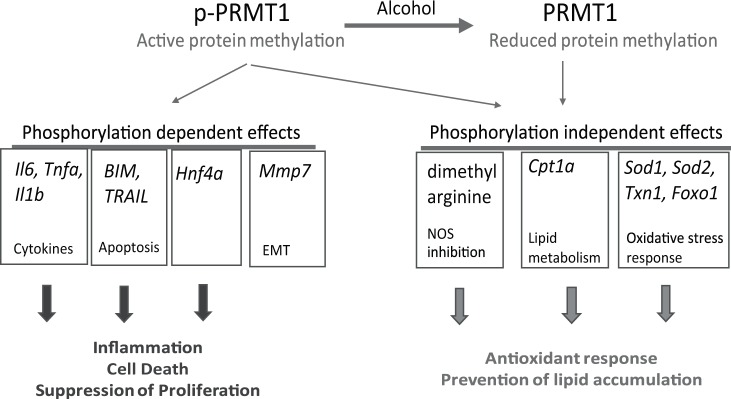
Model of PRMT1 activity shift in alcohol fed mice. There are two forms of PRMT1, the S297 phosphorylated form that predominates in the non-alcohol condition and a de-phosphorylated form that is generated after alcohol exposure. The phosphorylated form is more efficient in promoting the expression of cytokines, HNF4α and MMP7, BIM and TRAIL expression. Phosphorylation independent functions include Cpt1 expression, ADMA generation and the antioxidant stress response.

In summary we found that PRMT1 protects the liver from alcohol by both activity-dependent and activity-independent mechanisms. Previously we showed that PRMT1 is upregulated shortly after alcohol exposure^14^. In addition, here we found that there are two forms of PRMT1, the S297 phosphorylated form that predominates in the non-alcohol condition and a de-phosphorylated form that is generated after alcohol exposure (Fig. 7). The phosphorylated form is more efficient in promoting the expression of cytokines, HNF4α and MMP7, BIM and TRAIL expression. Phosphorylation independent functions include Cpt1 expression, ADMA generation and the antioxidant stress response. Alcohol dephosphorylates PRMT1, thus promoting its shift to become a factor that promotes a less pro-inflammatory, more pro-proliferative and pro-survival hepatocyte phenotype in the presence of alcohol. Failure to dephosphorylate PRMT1, as seen from S297D mimic experiments, results in higher susceptibility to oxidative stress induced apoptosis. These results suggest that PRMT1 is an important factor of liver adaptation to alcohol.

Our results have important therapeutic implications. PRMT1 has a well-established role in inflammation and cancer^23,25,26,45–49^; and there are ongoing pre-clinical studies investigating the potential of PRMT1 inhibitors (AMIs) in treatment^46,48,50^. However, our results indicate that in the liver PRMT1 is necessary for alcohol protection and PRMT1 induction in this case might be beneficial. Betaine supplementation has previously been demonstrated to protect the liver against alcohol. Betaine is required for the generation of methionine from homocysteine, a reaction that is central to the recycling of S-adenosyl-L-methionine (SAM), the methyl group donor for PRMT1. Some studies suggest that beneficial effects of betaine and SAM supplements are in part due to PRMT1 activation^51,52^. Other factors known to promote PRMT1 expression include IL4, TXNIP and PDGF^18,53,54^, however their role in alcohol pathogenesis is not yet evaluated. Future studies are necessary to determine the potential of inducing PRMT1 in the livers of patients with alcoholic liver disease.

## Materials and Methods

All methods were performed in accordance with the relevant guidelines and regulations and approved by Institutional Animal Care and Use Committees, Human Subjects Committee and Biosafety Committee of the University of Kansas Medical Center.

### Mice

C57BL/6NTac-Prmt1^tm1a(EUCOMM)Wtsi^/WtsiCnbc mice were obtained from EUCOMM (EUCOMM project: 40181) and bred with Flp recombinase mice to get homozygous Prmt1 floxed breeders as described before^30^.

All mice were housed in a temperature-controlled, specific pathogen-free environment with 12-hour light-dark cycles and fed regular mouse chow and water ad libitum. All animal handling procedures were approved by the Institutional Animal Care and Use Committees at the University of Kansas Medical Center (Kansas City, KS).

### Antibodies used

#### Primary antibodies

Anti-PRMT1 (F339), anti-pSer MAPK/CDK substrate, anti-H4, anti-Foxo1, anti-FOXO3 (N-terminal), anti-SOD2 and anti-cleaved Caspase3 antibodies were from Cell Signaling. Anti-β-actin, anti-SOD1, anti-p300 antibodies were from Santa Cruz. Rabbit Anti-PRMT1 antibody (against aa 300–361), pan anti-pSer, anti-SAM68 antibodies, were from Abcam. Anti-asymmetric-dimethyl-arginine antibodies, and Anti-H4R3me2a antibodies were from ActiveMotif. Mouse anti- β-actin, mouse Monoclonal Anti-PRMT1 clone 171 (against aa 1–361), Anti-Flag antibodies were from Sigma-Aldrich, Saint Louis, MO.

#### Secondary antibodies

IRDye 800CW goat anti-mouse IgG and IRDye 680RD goat anti-rabbit IgG were from Li-COR. General HRP-conjugated secondary antibodies were from Southern Biotechnology Associates (Birmingham, AL).

### Cell culture

Huh7.5 cells^55^ (obtained from Dr. Charles Rice) and were maintained in Dulbecco’s Modified Eagle’s Medium (Invitrogen, Carlsbad, CA) containing 10% FBS, 50 U mL^−1^ penicillin and 50 mg mL^−1^ streptomycin. Cells were transfected using Lipofectamine 3000 transfection reagent (Invitrogen) according to the manufacturer’s protocol.

### Vectors

pCMV6-PRMT1 vector was from Origene. The PRMT1 point mutations were generated by site directed mutagenesis (Quickchange kit, Stratagene). AAV8.TBG.PI.Null, AAV8.TBG.PI.Cre, AAV8-U6-JMJD6shRNA and AAV8-U6-ScrambledshRNA were from Vector BioLabs, Malvern, PA.

### Human specimens

De-identified human specimens were obtained from the Liver Center Tissue Bank at the University of Kansas Medical Center. Informed consent was obtained from all study participants. All studies using human tissue samples were approved by the Human Subjects Committee of the University of Kansas Medical Center.

### Real time PCR

RNA was extracted from cultured cells using the RNeasy Mini Kit (Qiagen). cDNA was generated using the RNA reverse transcription kit (Applied Biosystems, Cat. No. 4368814). Quantitative real time RT-PCR was performed in a CFX96 Real time system (Bio-Rad) using specific sense and antisense primers combined with iQ SYBR Green Supermix (Bio-Rad) for 40 amplification cycles: 5 s at 95 °C, 10 s at 57 °C, 30 s at 72 °C.

Primers were as follows: Gapdh, cgtcccgtagacaaaatggt, ttgaggtcaatgaaggggtc; Tnfa, aggctctggagaacagcacat, tggcttctcttcctgcaccaaa; CyclinB1, cagagttctgaacttcagcctg, ttgtgaggccacagttcaccat; p21, gcagatccacagcgatatcc, acaccagagtgcaagacagc; Prmt1, aacatgcagaggatgccagt, actccatgtttcacaatcggca; Nrf2, tcttgcctccaaaggatgtca, atggacttggagttgccacc; Sod1, gggaagcatggcgatgaaag, aacacaactggttcaccgct; Sod2, gcctgctctaatcaggaccc, tagtaagcgtgctcccacac; Cat, gatctcggaggccataatccg, ccgaccagggcatcaaaaac; Txn1, acaccacattggaatacttgtcac, gtggtgtggaccttgcaaaa; Cpt1a, ggttaacagcaactactacgcc, cagctctcgctgcctgaata; Il6, ttccatccagttgccttctt, cagaattgccattgcacaac; Mmp7, ggcttcgcaaggagagatca, gccaaattcatgggtggcag.

### Western blots

Protein extracts (15 µg) were subjected to 10% SDS-polyacrylamide gel electrophoresis (SDS-PAGE), electrophoretically transferred to nitrocellulose membranes (Amersham Hybond ECL, GE Healthcare), and blocked in 3% BSA/PBS at RT for 1 hour. Primary antibodies were incubated overnight at manufacturer recommended concentrations. Immunoblots were detected with the ECL Plus Western Blotting Detection System (Amersham Biosciences, Piscataway, NJ) or using near-infrared fluorescence with the ODYSSEY Fc, Dual-Mode Imaging system (Li-COR). Additional exposure images are provided in Supplementary data. Expression levels were evaluated by quantification of relative density of each band normalized to that of the corresponding β-actin or GAPDH band density.

### Immunohistochemistry

Immunostaining on formalin-fixed sections was performed by deparaffinization and rehydration, followed by antigen retrieval by heating in a pressure cooker (121 °C) for 5 minutes in 10 mM sodium citrate, pH 6.0 as described previously^14^. Peroxidase activity was blocked by incubation in 3% hydrogen peroxide for 10 minutes. Sections were rinsed three times in PBS/PBS-T (0.1% Tween-20) and incubated in Dako Protein Block (Dako) at room temperature for 1 hour. After removal of blocking solution, slides were placed into a humidified chamber and incubated overnight with an antibody, diluted 1:300 in Dako Protein Block at 4 °C. Antigen was detected using the SignalStain Boost IHC detection reagent (catalogue # 8114; Cell Signaling Technology, Beverly, MA), developed with diaminobenzidene (Dako, Carpinteria, CA), counterstained with hematoxylin (Sigma-Aldrich), and mounted. Signal intensity was analysed by Aperio ImageScope 12.1.

### Proximity ligation assay

Proximity ligation assays (PLA) were carried out using PLA kit (Sigma) according to manufacturer’s instructions. Following treatment cells were fixed with 4% PFA, washed and permeabilized with 1% Triton in PBS, blocked with supplied PLA blocking buffer and incubated with primary antibody against PRMT1, ubiquitin or pSer as indicated. Interactions were visualized using Duolink Brightfield detection reagent (Sigma). The PLA assay omitting one or both primary antibodies was used as a negative control. PLA signal was quantitated using Aperio ImageScope software.

### Immunofluorescence

Cells were fixed with 4% paraformaldehyde for 15 minutes at room temperature, washed with PBS and permeabilized with 1% Triton X-100 for 15 minutes then blocked in immunofluorescence buffer (PBS containing 2.5 mM EDTA, 1% BSA) for 1 hour. Cells were then incubated with primary antibody, 1:300 in PBS containing 2.5 mM EDTA, 1% BSA, 0.1% Triton X-100 overnight at 4 °C. After washing with PBS, coverslips were incubated with Alexa Flour -conjugated secondary antibody (1:500) in 0.1 µg/ml DAPI for 1 hour in the dark at RT. Coverslips were washed and mounted with FluorSave Reagent (Calbiochem. La Jolla, CA). Slides were observed in a Nikon Eclipse 800 upright epifluorescence microscope (Nikon Instruments, Melville, NY). Images were acquired using a Nikon CoolSNAP camera.

### Chromatin immunoprecipitation (ChIP) assay

Chromatin immunoprecipitation was performed as described previously^30,39^. Cells (1.5 × 10^7^) were cross-linked by the addition of 1% formaldehyde for 10 minutes. Cells were lysed with [10 mM Tris-HCl (pH 8.0), 10 mM NaCl, 3 mM MgCl2, 0.5% NP-40]. Nuclei were collected by centrifugation, resuspended in [1% SDS, 5 mmol/L EDTA, 50 mmol/L Tris-HCl (pH 8.0)] and sonicated to generate chromatin to an average length of ~100 to 500 bp. Next, samples in [1% Triton X-100, 2 mM EDTA, 20 mM Tris–HCl of pH 8.1, 150 mM NaCl], were immunoprecipitated overnight at 4 °C with 4 μg ChIP-grade antibody. 20 µl of magnetic beads (Dynabeads M-280, Invitrogen) were used to purify immunocomplexes. Following purification, cross-links were reverted by incubation at 65 °C for 6 h. Samples were purified with Qiagen kit.

Primers were as follows: Sod1, gggaactttctcagtccgca, gcgccacggagcttttatag; Sod2, cacgcggcctctaccaattt, ccgcaaggacacagcgaa.

### TUNEL assay

TUNEL assay was performed using the DeadEND Colorimetric TUNEL System (Promega) according to the manufacturer’s instructions.

### ADMA measurement

Serum ADMA was measured using universal ADMA ELISA kit (Novus) according to manufacturer’s instruction.

### Isolation of mouse primary hepatocytes

Primary hepatocytes were freshly isolated from mouse liver as described before^39^. Cells were isolated using a multi-step collagenase procedure as described in detail^56^. Media consisted of Williams’ Medium E (Life Technologies) supplemented with l-glutamine (2 mM) (Life Technologies), HEPES (10 mM), insulin (10^–7^ M), dexamethasone (10^−7^ M), penicillin (100 U/mL), streptomycin (100 μg/mL) and amphotericin B (0.25 μg/mL). The hepatocytes were brought to a concentration of 0.5 × 10^6^ cells/ml in Williams’ Medium E, as described above, plus 5% bovine calf serum. The hepatocytes were then seeded on collagen coated plates and allowed to attach in a humidified 37 °C, 5% CO2 incubator for 12 h and then treated as indicated.

### Statistics

Results are expressed as mean ± SD. The Student t test, paired t test, Pearson’s correlation, or one-way ANOVA with Bonferroni post hoc test was used for statistical analyses. p value < 0.05 was considered significant.

## Supplementary information

Supplemental data

## Data Availability

The data and materials generated during and/or analysed during the current study are available from the corresponding author on reasonable request.
