# Supplementary material for: Hepatocyte PRMT1 protects from alcohol induced liver injury by modulating oxidative stress responses
Source: Sci Rep. 2019 Jun 24;9:9111. doi: 10.1038/s41598-019-45585-2 (PMC6591482; doi:10.1038/s41598-019-45585-2)
Supplement: Supplementary file 1 — Supplemental data [file 41598_2019_45585_MOESM1_ESM.pdf]

Supplemental material

Hepatocyte PRMT1 protects from alcohol induced liver injury by modulating oxidative stress responses

Jie Zhao[1], Abby Adams[1,2], Steven A. Weinman[1,2] and Irina Tikhanovich[1]*

Figure 3C

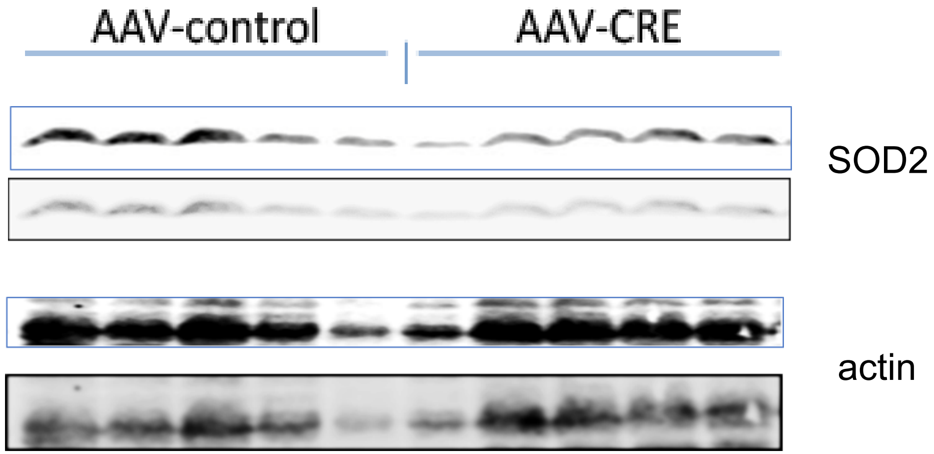

Figure 4A

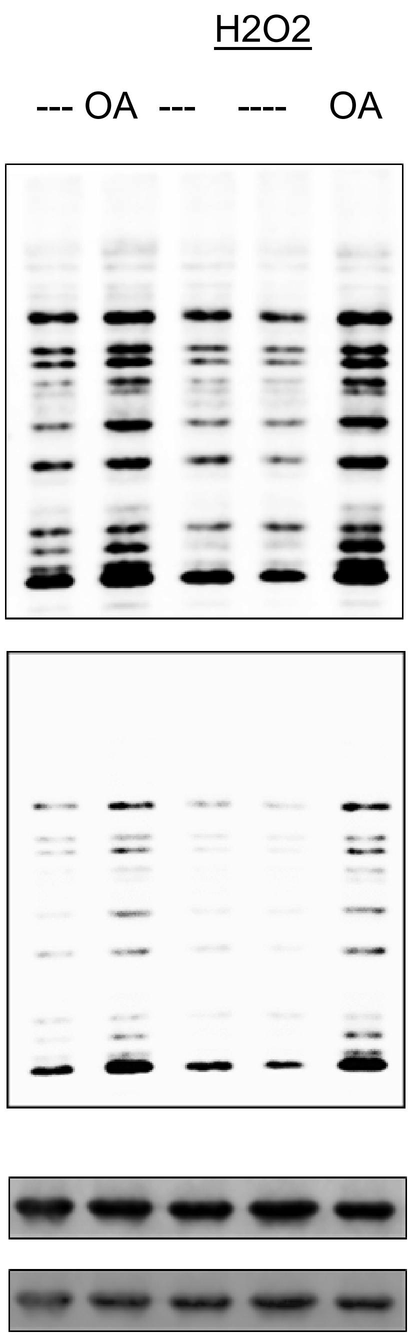

Figure 4D

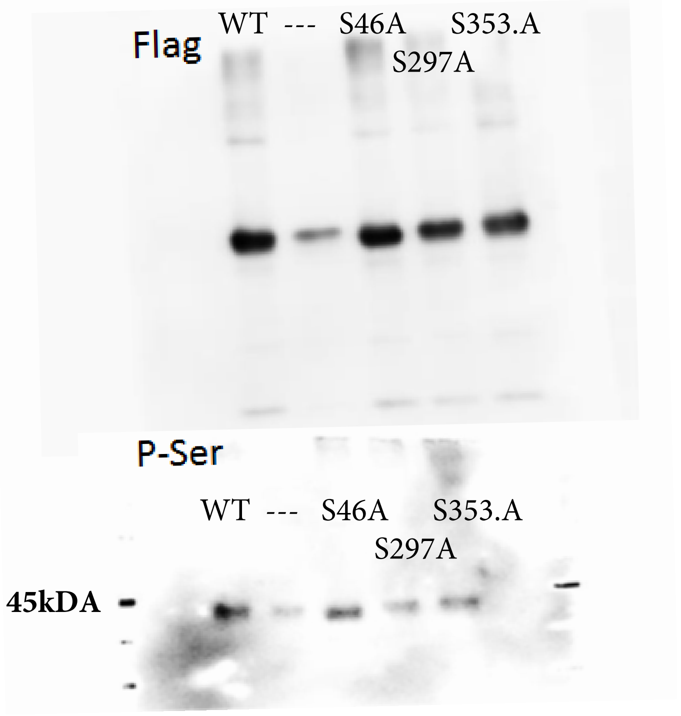

Figure 4F

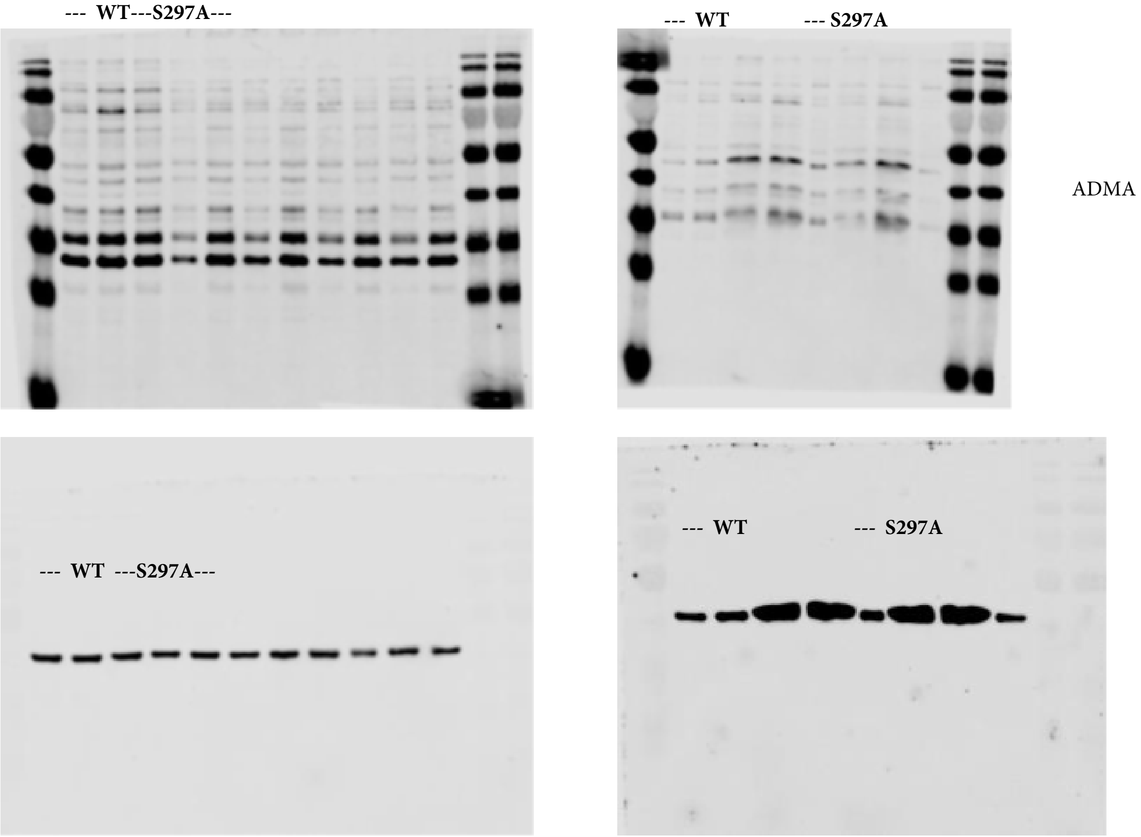

Figure 6E

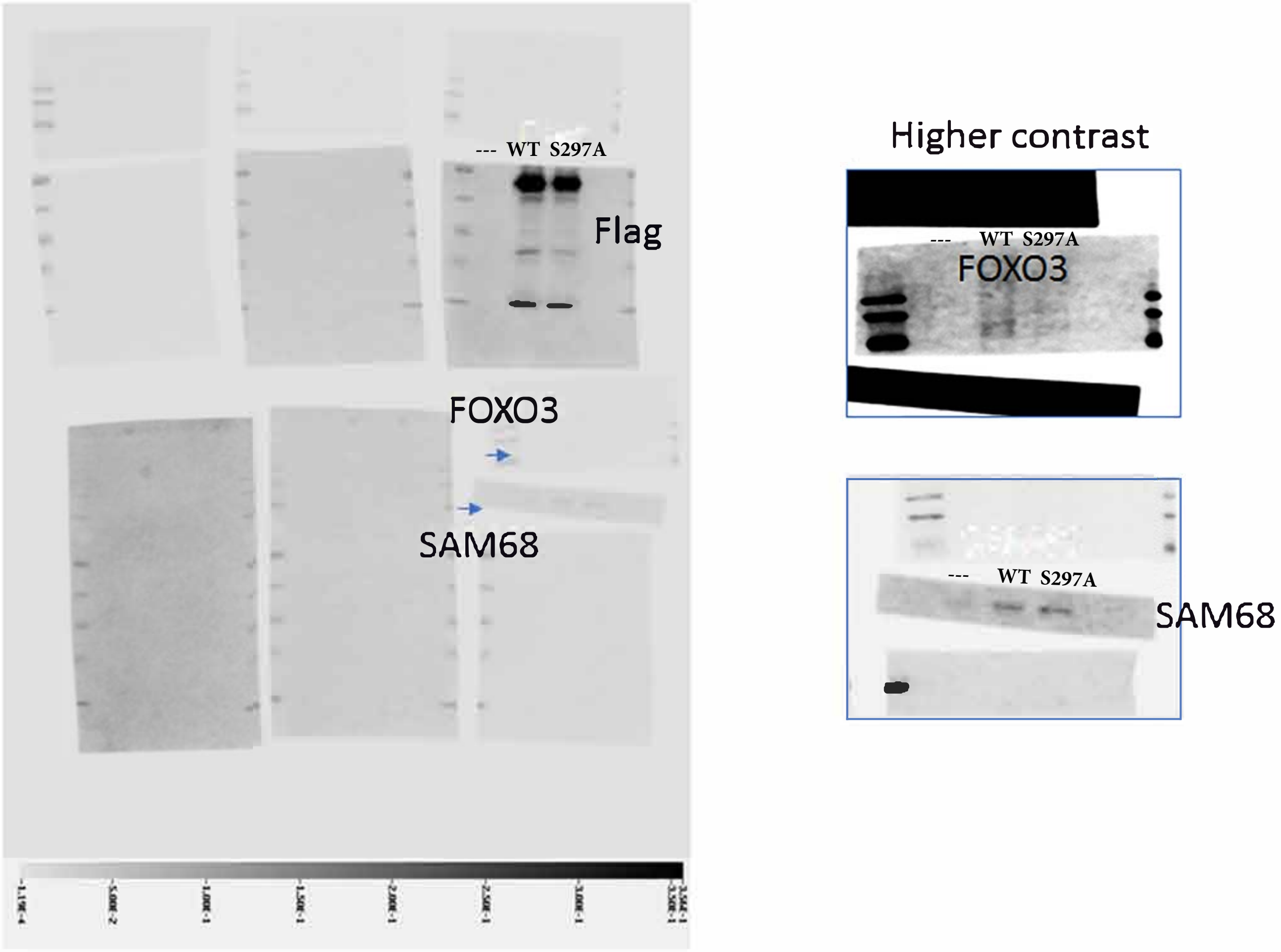

--- WT S297A
Flag
FOXO3
SAM68
Higher contrast
--- WT S297A
FOXO3
--- WT S297A
SAM68
